# Psychological Well-Being in a Connected World: The Impact of Cybervictimization in Children’s and Young People’s Life in France

**DOI:** 10.3389/fpsyg.2020.01427

**Published:** 2020-07-17

**Authors:** Catherine Audrin, Catherine Blaya

**Affiliations:** ^1^University of Teacher Education, Lausanne, Switzerland; ^2^Swiss Center for Affective Science, University of Geneva, Geneva, Switzerland; ^3^URMIS, Université Côte d’Azur, Nice, France

**Keywords:** cybervictimization, well-being, mental health, consequences, deviant behavior

## Abstract

The Internet is at the heart of our children’s and adolescents’ way of life. Although it opens up many positive perspectives in terms of access to information, knowledge, and communication, it also presents risks and potential negative experiences that can have severe consequences at the individual level. In this paper, we are interested in studying the link between cybervictimization, psychological well-being, and social competence. More specifically, we want to study how children and adolescents’ anxiety, impulsivity, self-esteem, and deviant behaviors may be related to cybervictimization. We collected data from 1019 children and young people in France aged 9–17 in the context of the EU Kids online survey. Sampling was performed building a random-probability nationally representative sample of households with children using the Internet. Participants completed a questionnaire online by computer-assisted self-interviewing (CASI). Structural equation model reveals that (1) cybervictimization is related to lower well-being, such as anxiety and low self-esteem, as well as lower social competence, such as impulsivity and deviant behaviors, and that (2) all dimensions of (non)well-being and social (in)competence are related to each other. Findings are discussed in the light of Agnew general strain theory and previous research findings on the consequences of cybervictimization.

## Introduction

The EU Youth Strategy states, “The health and well-being of young people should be supported, with a focus on the promotion of mental and sexual health, sport, physical activity and healthy lifestyles, as well as the prevention and treatment of injury, eating disorders, addictions and substance abuse.” Although well-being is largely influenced by socioeconomic conditions, findings from the OECD (2015) highlight that countries with similar levels of growth can have different well-being profiles and that there are gaps within countries and between different categories of population (youth–adults, men–women, etc.). Beyond economic factors, personal experiences, such as (cyber)bullying and violence, are related to young people’s well-being. Over the last decade, there has been a growing interest in the link between bullying, cyberbullying, and well-being; school climate, socioemotional development; and school achievement (O’Malley et al., 2012; Moisan, 2015; Shute and Slee, 2016; Poulin et al., 2018). Research highlights the importance of creating a safe and caring school environment and empowering students to build strategies to promote and safeguard their social and emotional well-being (Swearer et al., 2010).

The use of the Internet is worldwide and has become part of our daily lives. The 2015 PISA survey shows that 95% of 15-year-olds have Internet access at home. Young people are the most frequent users of the Internet and communicate on social media on a daily basis. In France, according to the latest Junior Connect 2018 survey on the digital practices of young people conducted by the IPSOS polling agency (4700 young people under 20), 13- to 19-year-olds spend more than 15 h a week on the Internet, and 7- to 12-year-olds about 6 h. Internet consumption increased by 45 min per day between 2015 and 2017 to the detriment of television even though it remains the main medium in terms of time spent on screens. It is, thus, important to understand how the use of such communication tools may be related to the well-being of children and young people.

As previously mentioned (Corcoran et al., 2015; Baldry et al., 2018), cyberbullying is related to a great variety of concepts and measures. Most authors agree on defining cyberbullying as (1) online aggressive and violent behaviors (2) that use electronic communication tools to bully others (Smith et al., 2013). However, some researchers consider cyberbullying to be the mere reproduction of bullying. In this conceptualization, cyberbullying refers to the intentional and repeated aggression over time within an imbalanced relationship between the victim and her/his aggressors using electronic tools of communications to perpetrate these aggressions (Smith and Steffgen, 2013). Others insist on the necessity to differentiate cyberbullying from cyberviolence, cyberstalking, cyberharassment, and cybervictimization (Wachs, 2012; Sticca et al., 2013; Corcoran and Mc Guckin, 2014; Blaya, 2015). In this paper, we refer to online victimization using the term “cybervictimization” as we did not include repetition and duration in our analyses and the intentionality is challenging to define.

Studies on French children and young people show a high prevalence of cyberaggression, amounting to up to 42% for cybervictimization and 6% for cyberbullying among 12- to 16-year-old respondents (Blaya and Fartoukh, 2015). This result is supported by Rémond et al. (2015), who interrogated 272 young people aged 16–18 and concluded that 35% of the respondents were victimized during the school year. Research has shown that cyberbullying is associated with many negative outcomes (Navarro et al., 2012; Álvarez-García et al., 2015) and that these outcomes can be both internalizing and externalizing problems (Hinduja and Patchin, 2019).

In the wake of the previous research mentioned above, we aim to assess how cybervictimization may be correlated with different aspects of well-being and social competence, such as how young people (1) feel anxious, (2) have low self-esteem (i.e., feel judged by others as being less intelligent and being less well treated by others), (3) are impulsive, and (4) adopt deviant behavior (i.e., tend to lie, steal, and fight). We also investigate how each type of the negative experiences mentioned above is related to each other.

## Research Background

### Psychological Well-Being and Social Competence

In this article, we draw from Houben et al. (2015) definition of well-being as they understand this concept as “a broad construct that involves either or both the presence of positive indicators of psychological adjustment such as positive emotionality, happiness, high self-esteem, or life satisfaction, and the absence of indicators of psychological maladjustment such as negative emotionality, psychopathological symptoms and diagnoses” (Houben et al., 2015, p. 901). This definition includes two main dimensions of well-being as they have been previously identified in the literature, namely the hedonic and eudaimonic dimensions. The hedonic part of well-being implies the maximization of positive affect and the minimization of negative affect (Disabato et al., 2016). Regarding the eudaimonic dimension, this refers to Aristotle’s work, in which well-being is more than happiness and pleasure, but instead regroups the capacity of being true to oneself and to evaluate one’s own functioning in life in order to work toward personal growth (Houben et al., 2015; Disabato et al., 2016). This definition highlights that well-being is related to the presence of positive elements (such as self-esteem) and the absence of negative elements (i.e., anxiety). Based on this definition, we consider well-being as including (1) high self-esteem and (2) absence of anxiety. High self-esteem is related to feelings of worth and self-respect (Rosenberg, 1965), and low self-esteem refers to feelings of self-rejection or self-contempt (Holopainen et al., 2012).

Although these variables are central on the individual level, we believe it is also important to consider variables related to social interactions and, more specifically, social competence (Romera et al., 2017). Social competence is defined as the capacity of interacting effectively with others (Rose-Krasnor, 1997). It comprises people’s impulsivity as well as disruptive or deviant behavior, reflecting some inability/difficulty to adapt in a relevant way to the dominant context (Holopainen et al., 2012). Impulsivity is defined as a difficulty to inhibit reactions, to wait for an outcome, and to plan ahead (Bear and Nietzel, 1991). Moreover, impulsivity manifests itself in terms of distractibility and high behavioral activity (White et al., 1994). Interestingly, there is scientific evidence that cybervictimization affects the victims’ capacities of concentration and, as a consequence, their academic performance (Baldry et al., 2018; Sorrentino et al., 2019). Although impulsivity is a risk factor for antisocial behavior, deviant behavior is directed toward others with a voluntary intention of annoying or harming them (Holopainen et al., 2012). As Kaplan reveals, people behaving disruptively or in a deviant way are teasing, annoying, or disturbing others (Kaplan et al., 2002). Literature suggests that low levels of social competence are related to antisocial behavior (Arce et al., 2011), whereas improving social competence may prevent involvement in cybervictimization (Gradinger et al., 2016).

### Cybervictimization and Well-Being of Children and Young People

Cybervictimization is correlated with negative outcomes for individuals that might be persistent and lifelong. Research shows that cybervictimization is related to increased internalizing (Lucas-Molina et al., 2018; Hinduja and Patchin, 2019) and externalizing negative behaviors and outcomes (Katzer et al., 2009; Sourander et al., 2010; Müller et al., 2018). Scholars have identified three major categories in terms of consequences: emotional and psychological consequences, academic and school-related consequences, and engaging in deviant behaviors.

Cybervictimization can be linked to emotional harm and high levels of mental health issues. This is particularly true when cybervictimization is performed using images and videos (Fahy et al., 2016; Yıldırım et al., 2017). It can also be related to lower self-esteem (Chang et al., 2013; Cénat et al., 2014; Tsaousis, 2016), high levels of anxiety (Campbell et al., 2012), distress, suicidal ideation and depression (Litwiller and Brausch, 2013; Rose and Tynes, 2015; Chu et al., 2018), alexythimia (Wachs et al., 2017), loneliness (Wright and Wachs, 2019), identity erosion, anger, fear, adopting violent behaviors, and suicide ideation (Tynes, 2005; Raskauskas and Stoltz, 2007; Didden et al., 2009; Blaya, 2010). Beyond the binary approach of victimized/not victimized, intersectional approaches focusing on bias (cyber)bullying show that minority students are more at risk of being cybervictimized. Research by Felmlee and Faris (2016) finds that homosexual and transsexual young people are four times more at risk of being cyberbullied than other young people. Minority groups are also subjected to more online hate both in the United States and in Europe (Llorent et al., 2016; Räsänen et al., 2016). Research shows that this type of aggression is motivated by prejudice toward individuals or communities and the victims’ real or supposed group/community membership (Poteat et al., 2014). This kind of aggression has even stronger negative effects. Wright and Wachs (2019) focus on the moderating effects of ethnicity on the consequences of cybervictimization and school attachment among seventh- and eighth-grade students. Their results highlight that Latinx respondents’ depression and anxiety levels were positively linked to cybervictimization and that they were strengthened by low levels of school attachment. On their side, Edwards et al. (2016) show that Latinx adolescent cybervictims revealed more suicidal ideation, depression, and suicidal behaviors than their Asian and Caucasian counterparts. Sexual minority youth are also notably vulnerable groups in terms of victimization (Kosciw et al., 2016; Elipe et al., 2018).

Literature further highlights that cybervictimization may be related to enhanced aggressive behaviors as well as internalizing and externalizing problems (Tsitsika et al., 2015; Fisher et al., 2016). In France, Kubiszewski et al. (2013) compared externalizing and internalizing consequences of cyberbullying and traditional bullying. Their findings show that cybervictimization has significant consequences in terms of mental health as cybervictims scored higher in terms of depressive feelings.

School-related consequences range from school avoidance (Payne and Hutzell, 2017), negative perceptions of school climate, decreased school well-being, and fear to go to school (Blaya, 2015) as well as reduced concentration capacities and lower academic achievement. Victimized youth may also be at increased risk of using substances, experiencing difficulties in school, participating in delinquent behavior, and engaging in unsafe sexual practices (Kowalski et al., 2014; Tsitsika et al., 2015). Goebert et al. (2011) and also Kowalski and Limber (2013) and Graham and Wood (2019) highlight that being cybervictimized is related to negative feelings among victims, such as anxiety or depression. Moreover, they show that cybervictimization is related to an increased probability to adopt deviant behaviors, such as self-harm, aggression, or delinquency. General strain theory of deviance (Agnew, 1992) shows how individuals can react to negative and stressful experiences or interpersonal relationships by adopting deviant behaviors. As cybervictimization is a negative interpersonal experience, we hypothesize that it is likely to be associated with deviant behaviors as a way of releasing stress caused by aggression or to cope with negative emotions.

### The Present Study

This paper is based on data collected as part of the EU Kids Online Survey. The purpose of this article is to study how psychological well-being and social competence are related to cybervictimization among young people in France. As the review of literature shows, previous research mostly focuses on internalizing dimensions of well-being and cybervictimization. In the wake of research proposed by Kubiszewski et al. (2013); Tsitsika et al. (2015), and Wright and Wachs (2019), we are interested not only in (internalizing) psychological well-being, but also in social competence and their link with cybervictimization. Results are drawn from a nationally representative sample in France, which was never performed before. We also examine which type of victimization (i.e., private or public victimization, online exclusion, online threat, or online compelling to do something) is most pernicious regarding both internalizing and externalizing dimensions of well-being. Our hypotheses are that different types of cybervictimization may be more strongly related to specific dimensions of young people’s well-being and social competence as suggested by Menesini et al. (2011), Ortega et al. (2012), or Mitchell et al. (2016). Ortega et al.’s (2012) research highlights that the emotional impact of cyberbullying depends on the type of cyberbullying. On their side, Mitchell et al. (2016) show that, when cyberbullying involves several perpetrators and is related to off-line events, it generates more severe psychological consequences. In the wake of these findings, our objective was to replicate similar research on a nationally representative sample in France.

## Materials and Methods

### Sample

The survey was completed by 1019 respondents aged 9–17 (Mean = 14.02, *SD* = 2.48). Slightly more than half of the sample were boys (*n* = 564, 55.34%). Most of the participants (*n* = 983, 96.4%) reported that French was the main language spoken at home. Table 1 shows the demographic characteristics of the participants. Sampling was performed building a random-probability nationally representative sample of households with children using the Internet. Participants were included on the basis of national data by the National Institute for Statistics for the following criteria: age of the child, sex of the child, region, urban/rural areas, parents’ occupation.

**TABLE 1:** Presentation of the sample.

	*N*	(%)
**Gender**		
Male	564	55.34
Female	455	44.65
**Main Language spoken at home**		
French	983	96.4%
Other	79	7.75%
**Parental occupation**		
Farmer	10	0.98%
Craftsmen, shopkeeper, business leader	89	8.73
Executives and senior professionals	204	20.02
Intermediate professions	227	22.27
Employees	180	17.66
Workers	240	23.55
Retired	22	2.15
With no activity	47	4.61
**Region**		
Urban	785	77.03%
Rural	234	22.96%

### Measures

We used the questionnaire built in the context of the EU Kids online survey. It is based on the questionnaire from the EU Kids online survey of 2010 and the Global Kids Online survey and was updated to meet the current evolutions of Internet use (Smahel et al., 2020). The full questionnaire in English and its national translations are available at www.eukidsonline.net. The questionnaire consisted of several groups of questions regarding (1) sociodemographic characteristics of participants, (2) their digital practices, (3) their experiences of bullying in schools as well as cybervictimization, (4) their experiences of cyberhate (i.e., exposure, victimization, and perpetration of hate online), (5) their attitudes toward religion, (6) their attitudes toward violence and racism, and (7) questions regarding their peer group. Participants further had to assess their level of psychological well-being (i.e., anxiety symptoms and self-esteem) as well as their social competence (i.e., impulsivity and deviant behavior). In this article, we focus on the questions referring to experiences of cybervictimization, well-being, and social competence.

Cognitive testing for 45 children aged 9–11 and 12–17 was performed to check and ensure comprehension and identify potential sources of measurement error. It was also piloted prior to the online survey to test the online implementation with 179 young people from the total age range of the sample in France.

#### Cybervictimization

Cybervictimization was measured by six items (α = 0.78, ω = 0.81). Participants were asked if, during the last 12 months, they (1) had received privately mean/nasty or unpleasant messages (item 1), (2) had someone publicly publish mean or unpleasant messages about them (item 2), (3) been left out or excluded from a group or activity on the Internet (item 3), (4) were threatened online (item 4), (5) were forced to do something they didn’t want to do (item 5), or (6) experienced other unpleasant or nasty things on the Internet (item 6). Participants could answer on a yes, no, I do not know scale. For the analyses, data were recoded as “1” for yes and “0” for no. Seventy-two participants (7.06% of the sample) selected the “I do not know” answer represented, and these values were considered as missing in the analyses.

#### Psychological Well-Being

Well-being was measured by two subdimensions. The first dimension was related to participants’ anxiety (α = 0.86, ω = 0.87). The five items of this dimension asked participants whether they had lots of worries, if they were often unhappy or sad, and if they were often scared. The second dimension consisted of four items measuring participants’ self-esteem (α = 0.85, ω = 0.86) with items such as “people think that you are not intelligent” and “other people seem to think that they are better than you.” For all these items, participants were asked to answer on a scale ranging from “1” (not true at all) to “5” (totally true).

#### Social Competence

Social competence was measured by two subdimensions related to (1) deviant behavior and (2) impulsivity (Holopainen et al., 2012). For each item, participants were asked to assess how each affirmation was true about them. The first dimension was designed to measure participants’ deviant behavior and consisted of five items such as “How true are these things about you: you get very angry and often lose your temper” (α = 0.69, ω = 0.77). The second dimension regrouped five items assessing a participant’s impulsivity, such as if they felt agitated, if they felt easily distracted, and if they thought a lot before doing anything (α = 0.69, ω = 0.86). For all these items, participants were asked to answer on a scale ranging from “1” (not true at all) to “5” (totally true).

### Procedure

Data was collected in June 2018 in the context of the EU Kids online survey (see Table 1). As the questionnaire was first designed in English, it was translated into French and then back into English to check on the validity and potential errors of translation. After piloting with all age ranges (*n* = 59), we decided to administer the same questionnaire to the younger and older participants as we did not identify any cognitive difficulty in the understanding of the questions or problems with the online survey. Data were collected by the OpinionWay polling agency, and the procedure complied with the national rules and procedure norm ISO 20252. Participants completed a questionnaire online by computer-assisted self-interviewing (CASI). The advantage of using an online questionnaire is that it allows a more playful visual layout for the young participants. In addition, because of the very personal nature of certain questions, the online self-administration method reduces the effects of social desirability and prevents respondents from feeling uncomfortable or judged by their responses.

Parents were asked to kindly keep away from their child while he/she was completing the survey. There was an adult referent from OpinionWay who could be contacted. The anonymity of participants was preserved, and all participants’ parents provided their active written consent.

Data were weighted for age and gender. Weighting was to meet the national representativity, and the margin of uncertainty was 1.5–3 points at the most for a sample of 1000 respondents.

### Data Analyses

Data were analyzed with R using the lavaan package (Rosseel, 2012) and consisted of three steps: descriptive statistics, structural equation modeling (SEM), and correlational analyses. We first report descriptive analyses on the prevalence of participants’ involvement in cybervictimization. In the SEM analysis, we tested how facing cybervictimization was related to specific dimensions of well-being (i.e., anxiety and self-esteem) as well as social competence (i.e., impulsivity and deviant behavior). We controlled for participants’ age, gender, and cyberaggression perpetration by introducing them as predictors of cybervictimization. Items were kept to define their latent factor if their loadings were equal or higher than 0.400. As most of our variables were categorical or ordered data, we used the WLSMV estimator. This estimator does not assume normally distributed variables and is recommended to analyze this kind of data (Brown, 2006). Finally, we were interested in examining how each item of cybervictimization is related with well-being and social competence. We, thus, aggregated anxiety, self-esteem, impulsivity, and deviant behavior and correlated them with each item of cybervictimization.

To assess the model’s goodness-of-fit, we relied on indices having different measurement properties as recommended by Hu and Bentler (1998). Thus, we used the root-mean-square error of approximation (RMSEA), the comparative fit indices (CFI), and the Tucker–Lewis index (TLI). Browne and Cudeck (1992) suggest that models with RMSEA below 0.05 are indicative of good fit and that values up to 0.08 reflect reasonable errors of approximation. The CFI statistic (McDonald and Marsh, 1990) reflects the “distance” of the model from the perfect fit. It is generally acknowledged that a value greater than 0.9 reflects an acceptable distance to the perfect fit. We also reported the TLI (Tucker and Lewis, 1973), which accounts for the model complexity. The TLI indicates how the model of interest improves the fit in relation to the null model. As for the CFI statistic, a TLI value equal to or greater than 0.9 reflects an acceptable distance to the perfect fit.

## Results

### Descriptive Statistics

Before analyzing our SEM results, we first provide descriptive analyses regarding participants’ experience of cybervictimization (Table 2). These results suggest that respondents were not very often victims of cybervictimization. However, such analysis highlights that the most frequent type of cybervictimization is receiving mean or insulting messages (12.48% yes) followed by being left out or excluded from a group or activity on the Internet (8.27% yes).

**TABLE 2:** Proportions of victims of cybervictimization.

Cybervictimization	No (%)	Don’t know (%)	Yes (%)
Item 1 Receiving privately mean messages	827 (85.78)	17 (1.76)	120 (12.44)
Item 2 Victim of online published mean messages	888 (91.92)	27 (2.79)	51 (5.27)
Item 3 Excluded from a group/activity online	868 (89.76)	19 (1.96)	80 (8.27)
Item 4 Threatened on Internet	911 (94.69)	14 (1.45)	37 (3.84)
Item 5 Forced to do something online	914 (95.01)	19 (1.97)	29 (3.01)
Item 6 Experience other mean things on Internet	909 (94.29)	18 (1.86)	37 (3.83)

We then report descriptive statistics of the items measuring participants’ well-being (Table 3: mean, standard deviation, skewness, and kurtosis) for victims (i.e., participants who responded at least once positively to the items presented in Table 1) and for the non-victim participants. As the scale was ranging from 1 to 5, results suggest that participants scored relatively low on these dimensions; however, victims tend to systematically score higher on these scales, revealing that they have lower levels of well-being and social competence than non-victim respondents.

**TABLE 3:** Descriptive statistics of well-being items for victims and non-victims of cybervictimization.

	Mean (victims, *n* = 185)	*SD*	Mean (non-victims, *n* = 834)	*SD*	Skew	Kurtosis
**Anxiety**						
Item 1 You worry a lot	2.36	0.98	1.85	1.00	0.51	−0.58
Item 2 You are nervous in some new situations, you easily lose confidence	2.32	1.02	1.80	1.02	0.65	−0.53
Item 3 You often have headaches, stomach aches or nausea	1.94	1.02	1.40	0.78	1.40	1.44
Item 4 You are often unhappy, sad or crying	1.87	0.96	1.40	0.74	1.27	1.27
Item 5 You have a lot of fears and you are easily scared	1.98	0.95	1.53	0.87	1.05	0.46
**Self-esteem**						
Item 1 Other young people/children are treated better than you	1.51	1.00	1.22	0.76	1.52	2.70
Item 2 People seem to think you’re not smart	1.64	0.93	1.24	0.74	1.54	2.44
Item 3 The others seem to think they’re better than you.	1.82	1.06	1.36	0.93	0.99	0.59
Item 4 The others give you mean nicknames or they insult you	1.56	0.87	1.24	0.70	1.99	4.04
**Impulsivity**						
Item 1 You’re agitated, you can’t stay still for very long.	1.95	0.98	1.57	0.91	1.04	0.13
Item 2 You finish the job you are given, you have a good ability to concentrate	2.36	1.01	2.56	1.14	−0.25	−0.96
Item 3 You’re always moving or squirming all the time	1.93	0.97	1.75	1.03	0.84	−0.35
Item 4 You are easily distracted and find it difficult to concentrate	2.39	1.03	1.82	0.99	0.59	−0.66
Item 5 You think before you do things	2.44	0.94	2.53	1.05	−0.24	−0.63
**Deviant behavior**						
Item 1 You get very angry and often lose your temper	2.00	1.06	1.61	0.92	0.99	0.08
Item 2 In general, you do what you are asked to do	2.62	0.94	2.58	1.04	−0.50	−0.49
Item 3 You fight a lot, you can make others do whatever you want.	1.43	0.88	1.16	0.57	2.40	6.58
Item 4 You are often accused of lying or cheating	1.64	0.90	1.29	0.67	1.70	2.76
Item 5 You take things that don’t belong to you at home, at school or elsewhere	1.49	0.85	1.15	0.57	2.59	7.07

### SEM Results

**FIGURE 1:**
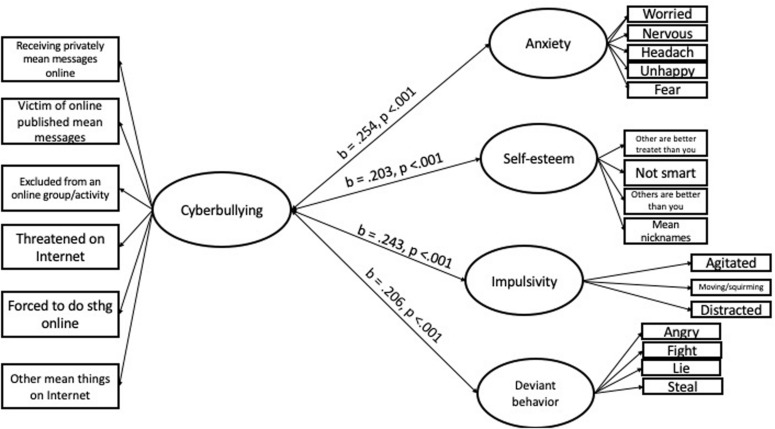
Results of the SEM model.

The model provided a good fit (RMSEA = 0.037, CFI = 0.993, TLI = 0.993, Chi^2^/df = 2.191). Graphical depiction is provided in Figure 1. Factor loadings are reported in Table 4 and correlations between latent factors in Table 5.

**TABLE 4:** Factor loadings.

Dimension	Standardized estimate	*SE*	Est/SE	*p*-value	Lower CI	Upper CI
**Anxiety**						
Item 1 You worry a lot	1.000	0.000			1.000	1.000
Item 2 You are nervous in some new situations, you easily lose confidence	1.166	0.044	26.25	0.001	1.079	1.253
Item 3 You often have headaches, stomach aches or nausea	1.064	0.049	21.91	0.001	0.969	1.159
Item 4 You are often unhappy, sad or crying	1.240	0.047	26.43	0.001	1.148	1.332
Item 5 You have a lot of fears and you are easily scared	1.021	0.047	21.56	0.001	0.928	1.114
**Self-esteem**						
Item 1 Other young people/children are treated better than you	1.000	0.000			1.000	1.000
Item 2 People seem to think you’re not smart	1.202	0.063	19.11	0.001	1.079	1.325
Item 3 The others seem to think they’re better than you	1.147	0.061	18.83	0.001	1.027	1.266
Item 4 The others give you mean nicknames or they insult you	1.196	0.073	16.27	0.001	1.052	1.340
**Impulsivity**						
Item 1 You are agitated, you can’t stay still for very long.	1.000	0.000			1.000	1.000
Item 3 You are always moving or squirming all the time	0.844	0.038	22.31	0.001	0.770	0.918
Item 4 You are easily distracted and find it difficult to concentrate	0.958	0.041	23.40	0.001	0.878	1.039
**Deviant behavior**						
Item 1 You get very angry and often lose your temper	1.000	0.000			1.000	1.000
Item 3 You fight a lot, you can make others do whatever you want.	0.816	0.056	14.56	0.001	0.706	0.926
Item 4 You are often accused of lying or cheating	0.985	0.045	22.02	0.001	0.898	1.073
Item 5 You take things that don’t belong to you at home, at school or elsewhere	0.960	0.051	18.76	0.001	0.860	1.061
**Cybervictimization**						
Item 1 Receiving privately mean messages online	1.000	0.000	NA		1.000	1.000
Item 2 Victim of online published mean messages	1.108	0.133	8.35	0.001	0.848	1.368
Item 3 Excluded from an online group/activity	0.986	0.132	7.46	0.001	0.727	1.245
Item 4 Threatened on Internet	1.208	0.158	7.63	0.001	0.898	1.519
Item 5 Forced to do something online	1.160	0.171	6.79	0.001	0.825	1.495
Item 6 Other mean things on Internet	1.247	0.162	7.72	0.001	0.930	1.564

**TABLE 5:** Correlation between latent factors.

Latent factor correlations	Standardized estimate	*SE*	Est/SE	*p*-value	Lower CI	Upper CI
Cybervictimization with anxiety	0.254	0.040	6.28	0.001	0.175	0.333
Cybervictimization with self-esteem	0.203	0.035	5.77	0.001	0.134	0.272
Cybervictimization with impulsivity	0.243	0.039	5.99	0.001	0.157	0.310
Cybervictimization with deviant behavior	0.206	0.038	5.42	0.001	0.132	0.281
Deviant behavior with anxiety	0.381	0.025	15.09	0.001	0.332	0.431
Deviant behavior with self-esteem	0.276	0.028	10.02	0.001	0.222	0.330
Deviant behavior with impulsivity	0.458	0.028	16.48	0.001	0.403	0.512
Anxiety with self-esteem	0.249	0.025	10.12	0.001	0.201	0.297
Anxiety with impulsivity	0.320	0.027	11.77	0.001	0.267	0.373
Impulsivity with self-esteem	0.280	0.028	10.03	0.001	0.226	0.335
Age on cybervictimization	0.040	0.021	1.91	0.056	−0.001	0.082
Gender on cybervictimization	0.178	0.094	1.90	0.058	−0.006	0.362
Cyberaggression on cybervictimization	1.153	0.309	3.73	0.001	0.547	1.759

Regarding the model *per se*, after controlling for gender (*b* = 0.178, 95% CI = [−0.006; 0.362], *p* = 0.058), age (*b* = 0.04, 95% CI = [−0.001; 0.082], *p* = 0.056) and cyberaggression perpetration (*b* = 1.153, 95% CI = [0.547; 1.759], *p* = 0.001), results reveal that being a victim is positively related to anxiety (*b* = 0.254, 95% CI = [0.175; 0.333], *p* = 0.001), self-esteem (*b* = 0.203, 95% CI = [0.134; 0.272], *p* = 0.001), impulsivity (*b* = 0.234, 95% CI = [0.157; 0.31], *p* = 0.001), and disruptive behavior (*b* = 0.206, 95% CI = [0.132; 0.281], *p* = 0.001). Together these results suggest that the more people report being victims, the lower they score on well-being and social competence.

Regarding correlations between specific negative dimensions of well-being and social competence, results reveal that all latent factors were positively and significantly related. More specifically, results show a significant and positive correlation between disruptive behavior and impulsivity (*b* = 0.458, 95% CI = [0.403; 0.512], *p* = 0.001), anxiety (*b* = 0.381, 95% CI = [0.332; 0.431], *p* = 0.001), and self-esteem (*b* = 0.276, 95% CI = [0.222; 0.33], *p* = 0.001). Results further highlight a positive correlation between anxiety, self-esteem (*b* = 0.249, 95% CI = [0.201; 0.297], *p* = 0.001), and impulsivity (*b* = 0.32, 95% CI = [0.267; 0.373], *p* = 0.001). Finally, results reveal a significant positive correlation between impulsivity and self-esteem (*b* = 0.28, 95% CI = [0.226; 0.335], *p* = 0.001).

### Correlational Analyses

Correlations are presented in Table 6 below. These reveal that all dimensions of cybervictimization are strongly related to all dimensions of well-being as well as social competence. Interestingly, items are particularly related to deviant behaviors (all *r* > 0.200), and this is especially true for people who were forced to do something online (*r* = 0.288, *p* < 0.001) or who suffered from mean things on the Internet (*r* = 0.263, *p* < 0.001). This means that the more people were forced to do something online and the more they suffer from mean things on the Internet, the more deviant behaviors they had. Being forced to do something online and suffering from things online were also related to low self-esteem (*r* = 0.264, *p* < 0.001; *r* = 0.272, *p* < 0.001, respectively) as well as being threatened on the Internet (*r* = 0.244, *p* < 0.001). Finally, receiving mean messages is positively related to anxiety (*r* = 0.232, *p* < 0.001).

**TABLE 6:** Correlation between cybervictimization items and aggregated latent factors.

	Deviant behavior	Anxiety	Impulsivity	Self-esteem
Item 1 Receiving privately mean messages online	0.210***	0.232***	0.151***	0.178***
Item 2 Victim of online published mean messages	0.212***	0.176***	0.124***	0.178***
Item 3 Excluded from an online group/activity	0.201***	0.197***	0.145***	0.179***
Item 4 Threatened on Internet	0.231***	0.196***	0.123***	0.244***
Item 5 Forced to do something online	0.288***	0.207***	0.193***	0.264***
Item 6 Other mean things on Internet	0.263***	0.213***	0.126***	0.272***

***p < 0.001.

These elements suggest that the coercive and threatening dimensions of cybervictimization are negatively related to youth’s deviant behavior and self-esteem.

## Discussion

This paper aimed to study how psychological well-being and social competence are related to cybervictimization of young people in France. To our knowledge, there was no other similar study based on a nationally representative sample in this country.

Descriptive statistics show that although victims were not that numerous, more than one respondent in 10 (12.5%) had received mean or insulting messages, and 8.27% were ostracized from an online activity. Other types of victimization were marginal. The findings of our survey show that cybervictimization is associated with strong negative consequences, such as higher anxiety and lower self-esteem, confirming previous conclusions from research in France (Kubiszewski et al., 2013). Together, these results suggest that the more people report being victims, the higher they score on all the negative dimensions of well-being assessed in this paper. Moreover, cybervictimization is also correlated with lower social competence, such as impulsivity and deviant behavior. Our results meet previous evidence that cybervictimization is a major mental health hazard (Ortega et al., 2012; Kubiszewski et al., 2013; Kowalski et al., 2014; Lucas-Molina et al., 2018).

Most research has investigated internalizing consequences of cybervictimization among perpetrators, but little research has studied the association of externalizing behaviors with victimization except for victims becoming aggressors in turn (Ybarra and Mitchell, 2007; Fisher et al., 2016). Our survey shows that deviant behavior has a strong association with cybervictimization, compared to other dimensions of psychological well-being, such as anxiety. This suggests that cybervictimization is related to a higher extent to externalizing behaviors. This result is in line with Agnew’s strain theory (Agnew, 1992) that shows that negative interpersonal relations are correlated to the adoption of deviant or delinquent behaviors.

Agnew (1992) further highlights the complex relations between stressful experiences, negative emotions, and antisocial behaviors. This theory suggests that those who are the least likely to adopt deviant behaviors are the young people who benefit from a strong social support in a meaningful, significant relationship. This stresses the importance of supporting the young people and providing them with the opportunity to build positive interpersonal relationships. Chu et al. (2010) confirm the importance of perceived support in children and adolescents’ well-being. They further reveal that teacher and school personnel’s perceived support are the strongest sources of support, followed by family members.

Cybervictimization presents some specific characteristics compared to bullying that are likely to increase stress and psychological malaise. For instance, the permanence of humiliating or nasty online content and the difficulty to erase it as well as the dissemination capacities of the Internet and sometimes the public nature of aggression might be factors that increase the emotional impact of victimization. However, findings from Ortega et al. (2012) conclude that the emotional impact is stronger for victims of traditional bullying compared to cybervictimization. This highlights the need for further research based on a longitudinal approach as cybervictimization and bullying may have lifelong deleterious consequences as both types of victimization are strongly correlated to similar negative outcomes (Del Rey et al., 2012; Kowalski and Limber, 2013).

In terms of overall practical implications, our results indicate that cybervictimization is negatively related to young people’s well-being and social competence. From a school perspective, teachers could collaborate with counselors or school social workers in order to provide not only support and workshops to inform students on the psychological consequences of cybervictimization, but also to set up sessions to teach students how to build up their self-esteem, assertiveness, and overall psychological well-being. As shown by Lee et al. (2015) some of most effective interventions against victimization are emotional control training as well as peer counseling. This last suggestion would potentially not only contribute to an overall increase in well-being but also act as a protective factor (Zych et al., 2019) and strengthen resilience capacity.

Several limitations must be mentioned. The first limitation is that this data is strictly transversal. Although our analyses did not include any causation, we believe future study should focus on the causal link between cybervictimization, well-being, and social competencies. Notably, longitudinal data might provide rich insight into this causal link (e.g., Wright et al., 2018). Our study did not have such an objective as we could not survey the very same young people twice, and we could not make a comparison with the EU Kids Online III study as the questionnaire was changed. Changes did not allow any comparison that would meet rigorous scientific standards. However, this could be a very relevant development. Another limitation refers to the fact that we used self-reported questionnaires and asked, during the same sessions, participants to assess not only their victimization, but also how well they felt. This might have created higher correlations than what would have happened if these constructs were assessed separately. Moreover, we did not formally test the content validity of our scales. As such, some items belonging to different scales may actually share common variance and present overlap between the measured concepts. Finally, our questionnaire did not allow us to analyze potential differences between occasional cybervictimization and repeated cybervictimization in terms of frequency and duration. Thus, further research is needed (1) to establish causal links between cybervictimization, well-being, and social competence, including the frequency and duration of cybervictimization and (2) to analyze the differential impacts of specific types of cybervictimization as some previous research shows that emotional responses are linked to types of cybervictimization (Ortega et al., 2012).

## Conclusion

This paper focuses on cybervictimization and its negative links with psychological well-being dimensions and social competence in young victims in France. Our results reveal that the more young people report being victims, the lower their psychological well-being. They report higher levels of anxiety and lower self-esteem. This confirms previous evidence highlighting cybervictimization as a major mental health hazard and less subjective well-being (Valois et al., 2012; Kowalski et al., 2014).

Our results further reveal that cybervictimization is strongly related to lower social competence and, notably, to deviant behaviors. This enhances the understanding of cybervictimization as a life stressor and a risk factor for deviant behaviors in line with Agnew’s general strain theory. Future studies should focus on family, school, and teacher support as a way to protect and prevent young people from suffering from the negative impacts of cybervictimization as well as on the role of peer mediation to promote resilience (Hinduja and Patchin, 2017). Although we adopted a correlational approach, further investigation is needed to analyze specific impacts of different types of cybervictimization on psychological, emotional, and behavioral responses and differences in coping strategies.

## Data Availability Statement

The datasets generated for this study will not be made publicly available because the data is still being analyzed. Requests to access the datasets should be addressed to the corresponding author.

## Ethics Statement

The studies involving human participants were reviewed and approved by Comité d’Ethique pour les Recherches Non Interventionnelles (CERNI), Université Nice Sophia Antipolis, Campus Valrose, 28 avenue Valrose, 06000 Nice. Written informed consent to participate in this study was provided by the participants’ legal guardian/next of kin.

## Author Contributions

CB designed the study. CA analyzed the data. Both authors contributed to the manuscript first draft as well as its revisions and read and approved the submitted version.

## Conflict of Interest

The authors declare that the research was conducted in the absence of any commercial or financial relationships that could be construed as a potential conflict of interest.
